# Evaluation of serum-based real-time PCR to detect *Schistosoma mansoni* infection before and after treatment

**DOI:** 10.1186/s40249-020-00698-z

**Published:** 2020-06-22

**Authors:** Antje Fuss, Humphrey Deogratias Mazigo, Andreas Mueller

**Affiliations:** 1 Medical Mission Institute, Hermann-Schell-Str. 7, 97074 Wuerzburg, Germany; 2 School of Medicine, Department of Medical Parasitology & Entomology, Catholic University of Health and Allied Sciences, P.O. Box 1464, Mwanza, Tanzania; 3 Klinikum Wuerzburg Mitte gGmbH, Medical Mission Hospital, Dept. of Tropical Medicine, Wuerzburg, Germany

**Keywords:** *Schistosoma mansoni*, Real-time PCR, Circulating DNA, Tanzania

## Abstract

**Background:**

To detect acute schistosomiasis, low-intensity infections, or to verify the success of treatment with praziquantel, highly sensitive test methods are required. The aim of this study was therefore to demonstrate the performance of *Schistosoma mansoni* specific DNA detection in serum and urine using real-time polymerase chain reaction (PCR) in an endemic area before and after treatment.

**Methods:**

The study pursued a 1-week and 20-weeks longitudinal design with a treatment intervention among 36 study participants aged 18 to 70 years in the community of Kayenze, a fishing village in Ilemela district on the southern shore of Lake Victoria in north-western Tanzania between February and June 2018. Blood, urine and stool samples were collected from each participant to diagnose *Schistosoma mansoni* infection before and two times after treatment with praziquantel using serum- and urine based real-time PCR, point-of-care circulating cathodic antigen (POC-CCA) rapid diagnostic test and the microscopic Kato-Katz (KK) method. Kappa coefficient (κ) was used to estimate the agreement between these diagnostic tests compared to a combined “gold standard” of positive results by serum-based real-time PCR and/or positive egg counts determined by KK. Kendall’s Tau rank correlation was used to examine the relationship between cycle threshold (Ct)-values and egg counts and the Wilcoxon signed-rank test was used to compare the median Ct-values of the different examination time points.

**Results:**

By using the combined “gold standard” of the parasitological Kato-Katz test and/or serum-based real-time PCR, a *S. mansoni* prevalence of 77.1% could be determined at baseline. In terms of sensitivity, serum-based real-time PCR (96.3%) and POC-CCA assay (77.8%) showed the highest results. The detection of DNA from urine samples showed the lowest sensitivity (33.3%). Treatment with praziquantel resulted in a significantly reduced prevalence of *S. mansoni.* No infection could be detected by Kato-Katz, with the POC-CCA test only 33.3%. The analysis of the median Ct values over time (which were determined by the serum-based real-time PCR) showed that the Ct decreases significantly shortly after treatment (from 30.3 to 28) and increases above baseline level (34.9) three months later.

**Conclusions:**

The data presented here show that the serum-based real-time PCR exhibits excellent diagnostic accuracy, in contrast to the use of urine as sample material for *S. mansoni* DNA detection. However, as circulating DNA does not necessarily reflect the persistence of living worms in schistosomiasis, this method is less well suited to verify the success of treatment with praziquantel.

## Background

Human schistosomiasis is considered as one of the most prevalent parasitic infections in the world with an estimated 229 million people requiring preventive treatment [1]. In sub-Saharan Africa, *Schistosoma mansoni* is one of the major species causing hepatic and intestinal schistosomiasis [2, 3]. The Kato-Katz method (KK) for microscopic detection of *S. mansoni* eggs in stool and the urine rapid diagnostic test for detection of circulating cathodic antigens (CCA) are extensively used for screening of schistosomiasis [4]. The KK method is cheap, easy to use and offers high diagnostic specificity in endemic areas with high parasite burden. However, sensitivity is low when this test is used in non-endemic areas or for the diagnosis of infections at an early stage [5]. The point-of-care circulating cathodic antigen (POC-CCA) test is also easy to use and the utilization of urine samples instead of stool as sample material leads to an increased acceptance by patients and a very good applicability for epidemiological surveys in large populations [6]. In numerous studies, especially with regard to *S. mansoni*, the POC-CCA test has shown higher prevalence rates than the KK test [7, 8]. Nevertheless, even this test seems to have limited sensitivity in areas with low infection intensity [9]. Therefore, both the microscopic KK test and the POC-CCA test are limited in terms of therapy control and assessment of control programs [10]. False negative results are a major problem, especially in elimination programs, because a limited number of surviving cases can spread infections rapidly and may negate the success of control programs [11, 12]. In addition, highly sensitive tests are important to detect acute schistosomiasis [13], low-intensity and residual infections [11, 12]. A suitable instrument for these applications could be molecular methods based on polymerase chain reaction (PCR) for the detection of parasite DNA. Parasitic cells or free-circulating parasitic DNA can be released from parasites into blood or urine and detected in these samples by PCR [14]. Thus, the use of stool samples, which often cannot be provided directly, can be avoided for the diagnosis of schistosomiasis [11]. The most desirable specimen is urine, as it is readily available, non-invasive, and can be easily collected in schools and clinics. However, patients are also often willing to provide a small amount of blood for examination [11]. Based on the frequently used sequence SM1–7, PCR [15], real-time PCR [16] and loop-mediated isothermal amplification (LAMP) [17] methods were established. SM1–7 is a highly repeated, tandemly arranged DNA sequence of *S. mansoni*, which contains 121 bp tandem repeats and comprises at least 12% of *S. mansoni* genome of both sexes [18]. Specificity tests have shown that there are no cross reactions with other helminths [18]. Wichmann et al. described in 2009 the detection of cell-free *S. mansoni* DNA by real-time PCR in plasma also using this sequence [19]. Due to the negative correlation between the cycle threshold (Ct)-value (defined as the number of cycles required for the fluorescent signal to exceed the background level) and the number of target copies, the real-time PCR method also offers the advantage of a quantitative analysis [20]. Therefore, this method might be suitable not only for the initial diagnosis but also for follow-up monitoring. First promising results were obtained in a previous study on experimentally infected mice [19].

In this study we investigated whether the detection of circulating free DNA from urine and serum samples using real-time PCR is suitable for diagnosing schistosomiasis compared to KK and POC-CCA test. In addition, we investigated whether serum-based real-time PCR can be used to assess the therapeutic success of praziquantel (PZQ) in a high prevalence setting on Lake Victoria. For this purpose, serum Ct values were compared during infection and after therapy.

## Methods

### Study area and population

The study pursued a longitudinal design with a treatment intervention and was conducted between March and July 2018 in the community of Kayenze, a fishing village in Ilemela district on the southern shore of Lake Victoria in north-western Tanzania. The majority of the inhabitants depend on the lake for domestic and economic activities including fishing, farming, washing, bathing, cooking, drinking and recreation. The region is endemic for *S. mansoni* [21–23] and the high occupational exposure keeps the intensity of *S. mansoni* infection high in adulthood [24]. Annual mass drug administration (MDA) using PZQ against schistosomiasis and albendazole against soil-transmitted helminthiases in this village is school-based and targets schoolchildren, not adults [21]. The study included only adult participants (aged 18–70 years) who provided written informed consent and had no treatment with PZQ in the last 12 months.

### Sample collection

Serum, urine and stool samples from adult participants with a proven *S. mansoni* infection were assessed during a baseline survey and again 1 week (6–8 days) and 20 weeks (142–144 days) after PZQ administration (40 mg/kg). These participants belonged to a larger group selected for a prevalence study. The cohort examined for the current study consisted of 36 participants, who had been present at all follow-up time points and of whom serum and urine samples were available for real-time PCR analyses. The stool sample of one study participant was missing at baseline data collection. Serum samples were obtained after centrifugation of coagulated blood samples at 1200×*g* for 5 min. The supernatant was centrifuged for further 15 min at 3000×*g* and then stored frozen at -20 °C. The centrifugation steps were performed at room temperature. Aliquots of urine (15 ml) were preserved with Norgen® Urine Preservative (Thorold, Canada) for nucleic acid extraction and stored at room temperature until use.

### Microscopic examination

Stool samples were evaluated for the presence of *S. mansoni* eggs by the quantitative Kato Katz (KK) thick smear technique. For the KK method two thick smears were prepared from different parts of a single stool sample using a template of 41.7 mg (Vestergaard Frandsen, Lausanne, Switzerland), following a standard protocol [25, 26]. After 24 h, the smears were independently examined for *S. mansoni* eggs by two experienced laboratory technicians of the National Institute for Medical Research (NIMR). For quality assurance, 10% of the negative and positive KK thick smears were re-examined by a third laboratory technician.

### POC-CCA urine rapid diagnostic test

Urine samples were tested for CCA of *Schistosoma* by the POC-CCA cassette test according to the protocol and procedures described by the manufacturer (Rapid Diagnostics, Pretoria, South Africa). Trace readings were considered as positive test results.

### DNA extraction from serum and urine samples

DNA extraction from 2 ml serum and 4 ml preserved urine were performed using the QIAamp Circulating Nucleic Acid Kit according to the manufacturer’s suggestions (Qiagen, Hilden, Germany). DNA was stored at − 20 °C after extraction.

### Amplification by real-time PCR

Detection of cell-free *S. mansoni* DNA samples was performed according to a previously published protocol [19] using a set of primers and probes complementary to a 121 bp tandem repeat sequence of *S. mansoni* strain SM 1–7 (GenBank accession number M61098) described by Hamburger et al. [18]. Primer sequences were: Sm FW 5′-CCG ACC AAC CGT TCT ATG A-3′; Sm RV 5′CAC GC TCT CG C AAA TAA TCT AAA-3′; Sm probe 5′-[FAM] TCG TTG TAT CTC CGA AAC CAC TGG ACG [(BHQ1])-3′ all synthesized by Eurofins Genomics, Ebersberg, Germany.

The 25 μl reaction mix contained 2.5 μl DNA, 1x QuantiFast Pathogen Master Mix (QuantiFast® Pathogen PCR + IC Kit, Qiagen, Hilden, Germany), 400 nmol/L of each Sm Primer and 200 nmol/L of Sm probe. The PCR runs consisted of an initial step of 5 min at 95 °C followed by 40 successive cycles of 15 s at 95 °C and 30 s at 60 °C. The reaction was run on the StepOne real-time PCR system (Applied Biosystems). DNA detection was expressed by Ct - values. In every run, the non-template control was negative (Ct = 0), the positive control (*S. mansoni* egg DNA) was positive and the exogenous Qiagen Internal Control to test successful amplification and to exclude the presence of PCR inhibitors was positive (Ct < 33) (The QuantiFast Pathogen PCR + IC Kit provides a ready-to-use Internal Control for universal use). A test was considered positive when the threshold was attained within 40 PCR cycles (Ct < 40).

### Statistical analyses

Statistical analysis were carried out using IBM SPSS Statistics version 24 (SPSS Inc., Chicago, USA) and Microsoft Excel 2013 (Microsoft Corporation, Redmond, USA). A *P*-value lower than 0.05 was considered statistically significant.

The prevalence of *S. mansoni* was determined for each diagnostic method and mean values (with 95% confidence intervals [CI]) and medians were calculated for the Ct-values and eggs per gram stool (EPG). This study used a combined diagnostic “gold standard” of positive results by serum-based real-time PCR and/or positive egg counts as determined by KK. A combined “gold standard” is used to obtain a reliable result and has been described in other studies [27]. Since this approach assumes a specificity of 100% for both test methods, only sensitivity and negative predicted value (NPV) were calculated for all assays used at baseline. Diagnostic results were converted to binary variables (1 = positive and 0 = negative). Kappa coefficient (κ) was used to statistically estimate the agreement between one diagnostic tool compared to the “gold standard”.

By using the real-time PCR method, the Ct-value is displayed. The Ct is defined as the number of cycles required for the fluorescent signal to cross the threshold (i.e. exceeds background level). Ct levels are inversely proportional to the amount of target nucleic acid in the sample (the lower the Ct level the greater the amount of target nucleic acid in the sample). The relationship between Ct-values and egg counts was examined by the Kendall’s Tau rank correlation (τ). A nonparametric test (Wilcoxon signed-rank test) was used to compare the median Ct-values of the different examination time points.

## Results

### Demographic information of study participants

Study participants who provided sample material for the current study consisted of 28 females (77.8%) and 8 males with a mean age of 34 years (range: 18 to 70 years).

### Baseline prevalence

Based on KK technique, the overall prevalence of *S. mansoni* was 34.3%. Egg loads varied between 12 and 1284 EPG with a median of 120 EPG. Real-time PCR detected *S. mansoni* DNA in 27 (75.0%) of serum samples with Ct-values ranging between 22.6 and 38 and a median Ct-value of 30.3. In urine, real-time PCR yielded 30.6% *S. mansoni* positive samples with Ct values between 33.9 and 40 and a median Ct-value of 38.5. The prevalence determined with the commercially available POC-CCA rapid test was 63.9% (Table 1).

**Table 1:** Percentages, mean (*CI*) and median (minimum, maximum) of positive results for the different diagnostic tests at baseline (pre-treatment) of the 36 study participants

Method	Cases/Total	%	EPG (Kato-Katz)/ Ct-values real-time PCR				
			Mean	*CI*	Median	Minimum	Maximum
**Microscopy (Kato-Katz)**	12/35	34.3	268	27.9–508.1	120	12	1248
POC-CCA	23/36	63.9					
**Serum real-time PCR**	27/36	75.0	30.4	28.8–31.9	30.3	22.6	38
Urine real-time PCR	11/36	30.6	37.7	36.3–39.1	38.5	33.9	40.7

*PCR* Polymerase chain reaction; *EPG* Eggs per gram; *POC-CCA* Point-of-care circulating cathodic antigen; *CI* Confidence intervals

### Assessment of the different diagnostic tools using a combined “gold standard”

The “gold standard” used was a combination of the parasitological KK test and/or real-time PCR of serum samples. According to this procedure, 77.1% (27/35) *S. mansoni* positive samples were determined. Due to a missing stool sample, only the results of 35 study participants could be included in the combined standard. The KK method showed a sensitivity of 44.4% with a negative predicted value (NPV) of 34.8%. Kappa agreement between the microscopy and the defined standard was poor (k = 0.268, *P* = 0.02). The POC-CCA test had a higher sensitivity (77.8%) and NPV (53.9%) than the KK test. The Kappa statistic was 0.535 (*P* < 0.001). Serum-based real-time PCR displayed the highest sensitivity (96.3%) and NPV (88.9%), as well as high Kappa agreement (k = 0.922, *P* < 0.001). This technique missed one out of 27 positive cases. The lowest sensitivity (33.3%) and NPV (28%) was achieved by the method of urine-based real-time PCR. The Kappa agreement between this test and the “gold standard” was 0.119 (*P* = 0.25). These results are summarized in Table 2. Due to the low performance of the urine-based real-time PCR, this method was not included in post-treatment examinations.

**Table 2:** Sensitivity, NPV with *CI* and Kappa statistic of the different diagnostic tests used at baseline. Serum-based real-time PCR and/or Kato-Katz results were used as “gold standard”

Test method	Sensitivity (95% ***CI***)	NPV (95% ***CI***)	Kappa (***P***)
**Microscopy (Kato-Katz)**	44.4% (25.5–64.7)	34.8% (16.4–57.3)	0.268 (*P* = 0.02)
**POC-CCA**	77.8% (57.7–91.4)	53.9% (25.1–80.8)	0.535 (*P* < 0.001)
**Serum-based real-time PCR**	96.3% (81.0–99.9)	88.9% (51.8–99.7)	0.922 (*P* < 0.001)
**Urine-based real-time PCR**	33.3% (16.5–54.0)	28% (12.2–49.4)	0.119 (*P* = 0.25)

*PCR* Polymerase chain reaction; *POC-CCA* Point-of-care circulating cathodic antigen; *NPV* Negative predicted value; *CI* Confidence intervals

### Post-treatment prevalence and intensity of *S. mansoni* infection

Treatment with PZQ resulted in a significantly reduced prevalence of *S. mansoni* after 20 weeks. Using the KK method, no infection could be detected (0%). The POC-CCA test detected 33.3% (12/36), the serum-based real-time PCR 58.3% *S. mansoni* positive samples (Table 3).

**Table 3:** Prevalences determined using the microscopic Kato-Katz method, the POC-CCA rapid diagnostic test and serum-based real-time PCR 20 weeks after therapy with praziquantel

Method	Cases/Total	%
**Microscopy (Kato-Katz)**	0/36	0
POC-CCA	12/36	33.3
**Serum real-time PCR**	21/36	58.3

### PCR polymerase chain reaction; POC-CCA point-of-care circulating cathodic antigen

Accordingly, serum-based real-time PCR showed the highest proportion of *S. mansoni* positives both at baseline and at 20 weeks after treatment. Examination of the median Ct-values, which are inversely proportional to the level of DNA, showed a median Ct value of 30.3 at baseline, which decreased significantly to 28 7 days after treatment (z = -2.395, *P* = 0.017). Twenty weeks after treatment, the median Ct-value increases significantly to 34.9 (z = -3.323, *P* = 0.001), and was significantly higher than baseline (z = -3.137, *P* = 0.002). The course of the Ct-values at the different examination time points is shown in Table 4. There was no significant correlation between the Ct values of the urine-based real-time PCR and the egg counts (τ = 0.098, *P* = 0.512) nor between the serum DNA levels and egg counts (τ = -0.032, *P* = 0.812).

**Table 4:** Proportion, mean (*CI*) and median (minimum, maximum) of positive results of the serum-based real-time PCR at the various examination time points

Serum real-time PCR	Pre-treatment	Post-treatment 1	Post-treatment 2
		(1 week)	(20 weeks)
**% positives**	27 (75)	27 (75)	21 (58.3)
Mean Ct-value (*CI*)	30.4 (28.8–31.9)	28.4 (27.1–29.7)	33.9 (32.2–35.5)
**Median Ct-value (min, max)**	30.3 (22.6, 38)	28 (21.5, 35.9)	34.9 (26.6, 38.6)

*CI* Confidence intervals; *PCR* Polymerase chain reaction; *Ct* Cycle threshold

## Discussion

In order to prevent egg-induced irreversible pathological reactions, highly sensitive tests for the diagnosis of schistosomiasis are of enormous importance. Hence, an ideal test should identify acute infections, low-intensity infections and residual infections, as well as allow assessment of treatment success and control programs. In this study, the value of detecting cell-free *S. mansoni* DNA in serum and urine was studied as an alternative diagnostic method to detecting eggs in stool or for detecting schistosome antigens in urine. The cell-free circulating schistosome DNA consists of fragments of parasite-derived DNA that is homogeneously distributed in plasma and other body fluids, such as urine or saliva of the host [15, 28–30]. Here, we were able to detect most infections with *S. mansoni* by serum-based real-time PCR (75%), followed by the POC-CCA test (63.9%). Comparably few infections were identified with the urine-based real-time PCR method (30.6%). To determine the diagnostic accuracy, the combined results of microscopy and serum-based real-time PCR were used as reference in this study, with both methods being considered 100% specific. This procedure has also been reviewed and applied in other studies [27, 31]. The serum-based real-time PCR performed excellently with 96.3% sensitivity, whereas the urine-based real-time PCR showed the lowest sensitivity (33.3%) and the lowest Kappa agreement with the “gold standard” (0.119). Various other studies indicate that the diagnosis of *S*. *mansoni* infection is possible using urine as a source of DNA [32–35]. In our study, the performance of the urine-based real-time PCR was even lower than the KK method, despite the fact that the DNA in the urine was stabilized with a special reagent. Maybe the preservation of DNA was not effective. Promising results were obtained from Lodh et al. with filtered urine samples [36, 37].

In addition, our results showed that the number of positives determined by serum-based real-time PCR was significantly higher 20 weeks after treatment than by parasitological stool examination. Using the KK method, no infection could be detected after treatment, as opposed to 58.3% with serum-based real-time PCR. There are several explanations for this discrepancy. First, this could be due to low sensitivity of the microscopy (44.4%) compared to the “gold standard” and could indicate that the egg-negatives were false negative. Second, reinfections with schistosomes are possible in high-transmission areas where humans are exposed to cercaria. The development from the schistosomula to the adult worm, which is capable of laying eggs, takes about 5–7 weeks [2]. In this prepatent period, where no eggs are detectable, schistosome DNA may be detected with the highly sensitive real-time PCR technique. However, since no eggs could be detected microscopically 20 weeks after treatment with PZQ, this indicates a rather slow dynamic of reinfection at this transmission site. Third, some authors point out that the effectiveness of praziquantel for the treatment of schistosomiasis is not as effective as previously thought, or that resistance may develop [28, 38–40]. This may indicate that real-time PCR can be a useful tool for evaluating treatment success. The last two mentioned aspects would also explain the 33.3% positive POC-CCA test results in our study. Lamberton et al. demonstrated in their drug efficacy study 2014, that the POC-CCA test was more sensitive than six KK slides at 4 weeks after PZQ treatment, and again at 6 months after chemotherapy [41].

In the current study, we were able to detect changes in the Ct value after treatment with PZQ and thus draw conclusions about changes of the amount of free circulating *S. mansoni* DNA. The free DNA presumably originates from the adult worms killed by the therapy [19], which leads to a significant increase in the DNA concentration in the hosts body (1 week post-treatment median Ct was 28). This was followed by an increase in the Ct value at the second follow-up examination (20 weeks after treatment median Ct was 34.9), which, was higher than at baseline (median Ct 30.3). Similar results have been reported in other studies [19, 42]. Since people with chronic infections have a large number of eggs in their tissues and DNA is probably released at very slow kinetics, free-circulating DNA may be detected several months after therapy [30, 43]. Nevertheless, studies on the detection of *S. japonicum* by classical PCR showed that the DNA of this parasite was no longer detectable 6–8 weeks after treatment with praziquantel [44]. It is also possible that the circulating DNA detected in this study, 20 weeks after treatment with praziquantel, is due to reinfection. Like many other studies, we have found no correlation between the Ct value and the number of eggs in the stool samples [45, 46]. One possible explanation for this is that the real-time PCR method used to detect *S. mansoni*-specific DNA is able to detect it from all stages of the life cycle. A positive PCR reaction shows that DNA of the parasite is present but not whether it comes from egg-producing worm pairs or, for example, larval stages [46].

## Conclusions

Despite the fact that the results of the current study are based on a very small sample size and this may have affected the results, we conclude that serum-based real-time PCR has an added value in a variety of situations. Although it cannot replace the KK method or the POC-CCA test in endemic areas, this method is well suited to detect schistosomiasis very early or infections with low intensities, because of its high diagnostic accuracy. This is particularly useful for travelers and migrants to prevent possible chronic complications of an unrecognized disease or for long-term monitoring of control interventions. However, as circulating DNA does not necessarily reflect the persistence of living worms in schistosomiasis, this method is less well suited to verify the success of treatment with praziquantel.

## Supplementary information

**Additional file 1.** Dataset

## Data Availability

All relevant data are within the paper and its Supporting Information files.

## Abbreviations

CCA: Circulating cathodic antigen
CI: Confidence intervals
Ct: Cycle threshold
KK: Kato-Katz
LAMP: Loop-mediated isothermal amplification
MDA: Mass drug administration
NIMR: National Institute for Medical Research
NPV: Negative predicted value
PCR: Polymerase chain reaction
POC-CCA: Point-of-care circulating cathodic antigen
PZQ: Praziquantel
